# Analysis of the Literature and Patents on Solid Dispersions from 1980 to 2015

**DOI:** 10.3390/molecules23071697

**Published:** 2018-07-12

**Authors:** Jinglu Zhang, Run Han, Weijie Chen, Weixiang Zhang, Ying Li, Yuanhui Ji, Lijiang Chen, Hao Pan, Xinggang Yang, Weisan Pan, Defang Ouyang

**Affiliations:** 1 State Key Laboratory of Quality Research in Chinese Medicine, Institute of Chinese Medical Sciences (ICMS), University of Macau, Macau 999078, China; zhangjinglu01@hotmail.com (J.Z.); hanrun1307@126.com (R.H.); mb55813@umac.mo (W.C.); 13980967387@163.com (W.Z.); 2 Department of Pharmacy, School of Medicine, Shenzhen University, Shenzhen 518060, China; 3 Jiangsu Province Hi-Tech Key Laboratory for Bio-medical Research, School of Chemistry and Chemical Engineering, Southeast University, Nanjing 211189, China; yuanhui.ji@seu.edu.cn; 4 School of Pharmacy, Liaoning University, Shenyang 110000, China; chlj16@163.com (L.C.); haopan0330@163.com (H.P.); 5 School of Pharmacy, Shenyang Pharmaceutical University, Shenyang 110000, China; yangxg123@163.com (X.Y.); ppwwss@163.com (W.P.)

**Keywords:** solid dispersion, big data, literature, patent, scientific knowledge mapping, CiteSpace

## Abstract

*Background*: Solid dispersions are an effective formulation technique to improve the solubility, dissolution rate, and bioavailability of water-insoluble drugs for oral delivery. In the last 15 years, increased attention was focused on this technology. There were 23 marketed drugs prepared by solid dispersion techniques. *Objective*: This study aimed to report the big picture of solid dispersion research from 1980 to 2015. *Method*: Scientific knowledge mapping tools were used for the qualitative and the quantitative analysis of patents and literature from the time and space dimensions. *Results*: Western Europe and North America were the major research areas in this field with frequent international cooperation. Moreover, there was a close collaboration between universities and industries, while research collaboration in Asia mainly existed between universities. The model drugs, main excipients, preparation technologies, characterization approaches and the mechanism involved in the formulation of solid dispersions were analyzed via the keyword burst and co-citation cluster techniques. Integrated experimental, theoretical and computational tools were useful techniques for in silico formulation design of the solid dispersions. *Conclusions*: Our research provided the qualitative and the quantitative analysis of patents and literature of solid dispersions in the last three decades.

## 1. Introduction

With the increasing discovery of water-insoluble Active Pharmaceutical Ingredients (APIs) high throughput screening, combinatorial chemistry and computer-aided drug design were extensively applied in formulation design since 1980 [1,2,3,4,5,6], the question of how to improve their poor water solubility has drawn more attention of the researchers than ever before. To date, various methods were reported to overcome the solubility issue of potential drug candidates at the formulation stage, such as prodrug formation [7], complexation [8], nano-suspensions [9], micelles [10], salt formation [11], particle size reduction [12], self-emulsifying drug delivery systems [13] and solid dispersions [14]. The solid dispersion technique was one of the most successful solutions in the pharmaceutical industry to increase the solubility of the poorly soluble drugs, used in over 15 marketed products [15]. The advantages of solid dispersion technology include reduction of the drug particle size, improvement in wettability, and crystal transformation to amorphous state [16]. Since the first solid dispersion in 1961 [17], this technology has been developed over 50 years by the date of this review. Currently, thousands of publications on solid dispersion are available and a significant increase in the number of research articles and patents has been observed in the past three decades. The focused issue of most of the above publications was the technical details of different formulations or new applications of this technology. However, there was a lack of qualitative and quantitative analysis of literature and patents published in this research field.

Mapping approach establishes bibliometric maps for the description of the concept formation, development and social structure of a specific research field or a scientific domain [18]. It could provide a special perspective to observe the research domain, which utilizes the big data analysis of a large amount of publications and patents. In comparison to traditional one-by-one reading strategy, mapping analysis can help the researchers to save large time and effort by the analysis of massive research literature and patents in the relevant field [19]. Currently, there are many software programs for the literature analysis, such as CiteSpace [20], VOSviewer [21,22], Sci2 [23], RefViz [19], HistCite [24], etc. For example, research on liposomes in global scale from 1995 to 2014 was analyzed by CiteSpace [25]. An analysis of nanoparticle drug delivery technologies from 2005 to 2014 was also done using CiteSpace [26]. Anti-diabetic drug research in China from 2009 to 2013 was analyzed by Gephi to visualize the network and relationships among institutes [27]. Publications and patents on natural anti-cancer drugs from 1990 to 2013 were analyzed using VOSviewer [28]. 

Due the increased interest in solid dispersions, this paper aims to provide a big picture of the solid dispersion area from 1980 to 2015 by analyzing the patents and literature in the research field of solid dispersions. The analytical topics included the characteristics of publication outputs or patent quantity, patent and publication distribution (e.g., countries, and research institutions), patentee or active researchers, global collaborations, main journals and the trend shift of the research frontier of literatures and patents.

## 2. Data Source and Analytical Methods

### 2.1. Data Source

The references data in this paper was obtained from Science Citation Index Expanded (SCIE) database and Derwent Innovation Index (DII) database via Web of Science. The data retrieval strategy was listed below:

For SCIE:
Topics = “solid dispersion” OR “solid dispersions”,
The publication period = “1980 to 2015”, (Retrieved date 30-04-2016).
For DII:
Topics = “solid dispersion*” AND
Derwent Manual Code = “B*”
The publication period = “1980 to 2015”, (Retrieved date 30-04-2016).

A total of 3451 publication records and 1076 patent records were obtained using the above parameters. The reference data from Web of Science included full records and cited references, such as articles’ titles, keywords, authors, source titles, affiliations, abstracts, publication date and citation number. The patent information included patent number, patentee, countries and abstracts.

### 2.2. Analytical Methods

CiteSpace, developed by Dr. Chaomei Chen at the College of Information Science and Technology, Drexel University (Philadelphia, PA, USA) [29,30,31], was selected as the main analytical software in this study. The network of cooperation among different countries and institutions was visualized using science bibliometric mapping. In addition, the keyword burst and co-citation were also investigated to explore the shift of research frontiers with the time.

The software parameters for CiteSpace were set up as follows:

(a) Years per slice: 6 years as the length of a single time slice;
(b) Threshold selection: the most cited references per time slice was selected to map the global cooperation of the top 50 countries and institutes; the references with the most citing numbers per time slice were selected to map the top 10% co-cited journals and literature.
(c) Pruning and merging: the pathfinder approach.

## 3. Result

### 3.1. Characteristics of Publication Output about Solid Dispersions from 1980 to 2015

**Figure 1:**
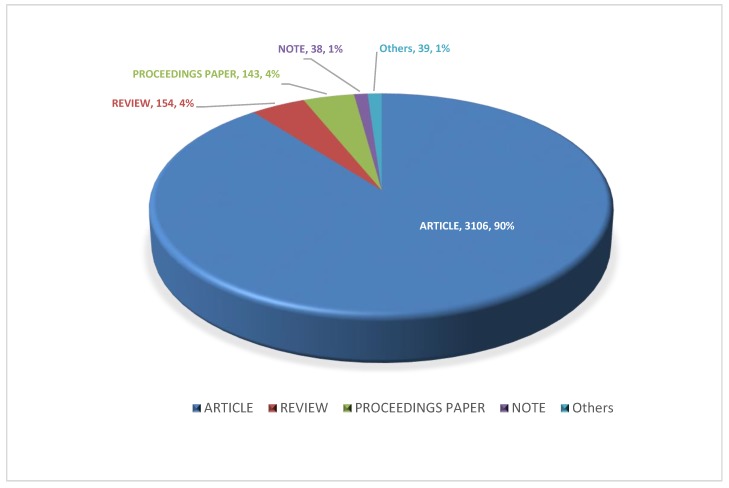
Categorization of solid dispersion publications by documents types from1980 to 2015.

3451 publications from past 36 years were classified into five different document types, including research articles, reviews, proceeding papers, notes and others. As shown in Figure 1, 3106 publications (about 90%) were research articles, follow by 154 reviews and 143 proceeding papers, both of which accounted for approximately 4%. The publication numbers for notes and others were 38 (1%) and 39 (1%), respectively.

**Figure 2:**
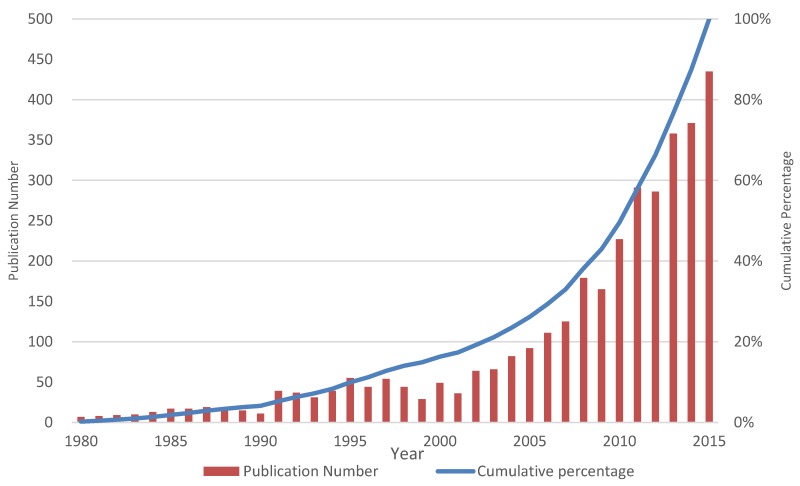
The publication number (red column) and cumulative percentage (blue curve) from 1980 to 2015.

Figure 2 shows the trend of the publication and patent number in the past three decades. The numbers for publications and patents increased dramatically after 2000, from 49 in 2000 to 435 in 2015, while global patent number increased from 11 in 2000 to 157 in 2015. More than 70% of publications and patents were published from 2005 to 2015, indicating that solid dispersion researches attracted comparatively more attention in the above period.

### 3.2. Analysis and Mapping of International Contribution

#### 3.2.1. Main Countries

The top ten most productive countries in publications and patents are listed in Table 1. These countries generated more than 80% of the publications and patents in the solid dispersion area. USA had the largest contribution in publication numbers, with a global share of 18.34%, followed by Japan (11.10%), China (10.72%), India (10.11%) and England (8.37%). Interestingly, Germany and Belgium only ranked 7th and 8th, but their average citation number per paper ranked in the top two, indicating a high research quality. China had the largest number of patent applications, 649, which was 147 more than that of the United States, 503. The third and fourth places were taken by the World Intellectual Property Organization (WO), 432, and the European intellectual property organization (EPO), 356, which is closely followed by Japan, 439.

**Figure 3:**
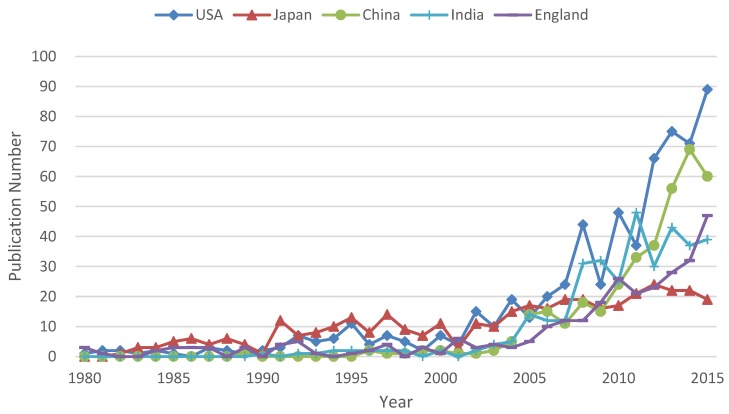
Annual publication numbers of top five productive countries.

Figure 3 presents the annual publication numbers of the top five countries in the previous 36 years. Japan held a leading position before 2000 and gradually increased in recent 15 years with smooth rate. USA and England were also the pioneers in this area, with a rapid growth after 2000. China and India were the late-comers in this field and the first publication records about solid dispersions in the WoS database were in 1989 (China) and 1990 (India), respectively. However, after 2005, there was a significant jump in China and India and they reached the 3rd and 4th position in 2015.

#### 3.2.2. Global Network of Cooperation

**Figure 4:**
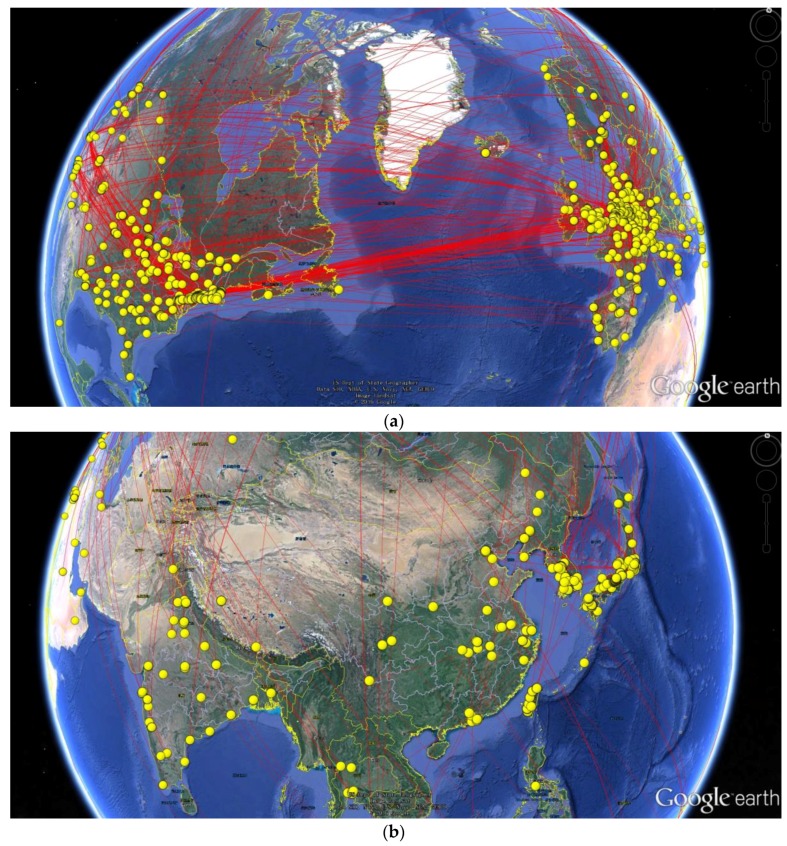
Global geographic distributions of authors according to the total number of publications by country: (**a**) North America and Europe; (**b**) Asia. Each yellow node showed individual researches and one red link between two nodes represented the collaboration at one publication.

**Figure 5:**
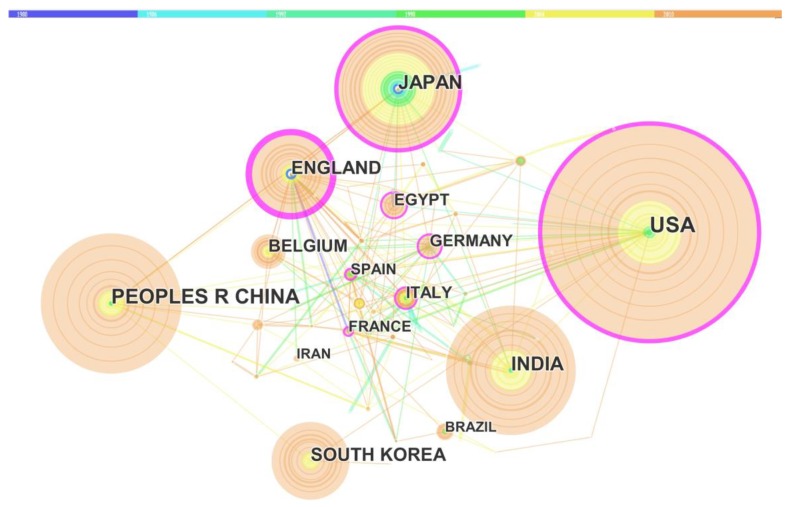
Country collaboration relationships network. Different colors in the figure represent different time slices, deep blue: 1980 to1985, sky blue: 1986 to 1991, pink green: 1992 to 1997, green: 1998 to 2003, yellow: 2004 to 2009 and orange: 2010 to 2015. The diameter of each circle represents the publication number of the country. The thickness of the connect line represents the intensity of country cooperation.

CiteSpace and Google Earth programs were used to visualize the global network of cooperation relation distribution, which offered direct observation of the international cooperation from a broad perspective. As shown in Figure 4, the authors’ affiliations were mapped worldwide to investigate collaborations. According to Figure 4a, the cooperation clusters were mainly located within the Western Europe and North America as showed the strong intensity of cooperation in these areas. Figure 4b showed the collaboration within Asia, which focused on Japan and South Korea. Although both China and India had high numbers of publication, the international collaboration was much less than other countries. Figure 5 presented the network of collaboration among different countries. The purple circle represented the intensity of international collaboration networks, while the direct line plays as a bridge role in the international cooperation. It clearly showed that England, Japan, USA, Italy and Germany were the top five countries in international cooperation, which was in agreement with the institutional cooperation. In addition, an increase of the global cooperation in this research field was observed during the past 30 years.

### 3.3. Analysis and Mapping of the Institution Distribution

The active research institutions and patent holders in this research area are listed in Table 2 and Table 3, respectively. Purdue University (West Lafayette, IN, USA), with 107 publications, was the top one, followed by Catholic University of Louvain (Leuven, Belgium) (92) and Shenyang Pharmaceutical University (Shenyang, China) (73). 

It is worth noting that many famous pharmaceutical companies, such as Johnson & Johnson, Novartis, Roche Holding, AstraZeneca and Bristol Myers Squibb Co., were also very active in this area. The average citation number of the publications from Johnson & Johnson was 37.42 which ranked the first among commercial companies, the representative product of which using the solid dispersion technology was Incivek^®^. Moreover, Table 3 showed that most of the patent holders were commercial companies, which indicated that this technology was not only a basic research, but also a widely accepted and used technique in the pharmaceutical industry. This technique attracted great attention from pharmaceutical industry because the simplicity of formulations and manufacture process of solid dispersions made the poorly soluble drugs to be easy commercialization and over 15 products had been approved by the FDA [32].

#### 3.3.1. Network of the Institution Cooperation

**Figure 6:**
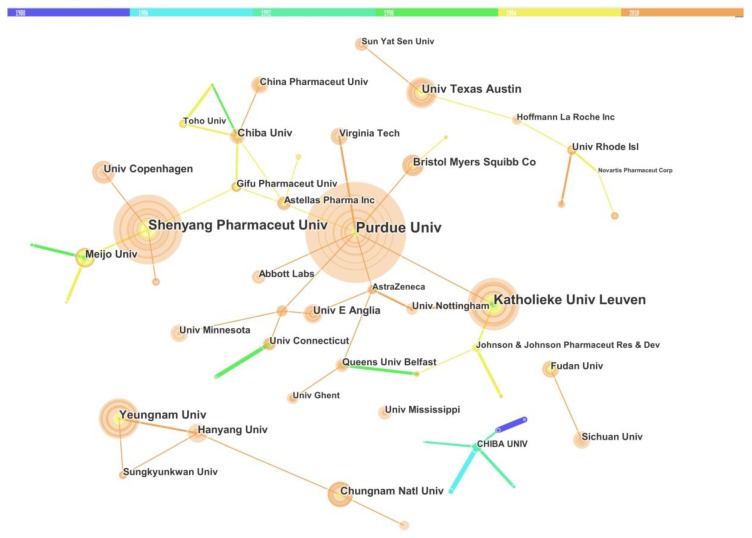
Mapping of Institution cooperation network. Different colors represent different time slices, deep blue: 1980 to1985, sky blue: 1986 to 1991, pink green: 1992 to 1997, green: 1998 to 2003, yellow: 2004 to 2009 and orange: 2010 to 2015 from 1980 to 2015. The diameter of each circle represents the publication number of the institution. The thickness of the connect line represents the intensity of institution cooperation.

Figure 6 shows the analysis of institution cooperation in the solid dispersion field. The number of yellow lines (2004 to 2009) and orange lines (2010 to 2015) were much more than the deep blue (1980 to 1985) and green lines (1998 to 2003), indicating a stronger cooperation between organizations in recent years. This was also in agreement with the data of country cooperation as presented in Figure 5. One possible reason was that the rapid development of information and communication technology made the cooperative research much easier than ever before [33,34]. Interestingly, the frequent cooperation between universities and pharmaceutical companies was observed in the USA and Europe. For example, Purdue University had wide links with many pharmaceutical companies, such as Abbott Lab., AstraZeneca, Astellas Pharma Inc. and Bristol Myers Squibb Co. These leading giants in the pharmaceutical industry worked together with research institutions to establish large research clusters. The collaboration could effectively reduce the high cost of research and development (R&D) and improve the research efficiency [35,36]. Meanwhile, there were more academic collaborations within Asian universities. For example, Shenyang Pharmaceutical University had a wide collaboration with different universities, such as Meijo University, Gifu Pharmaceutical University and the University of Copenhagen. 

#### 3.3.2. Network of Authors’ Cooperation

**Figure 7:**
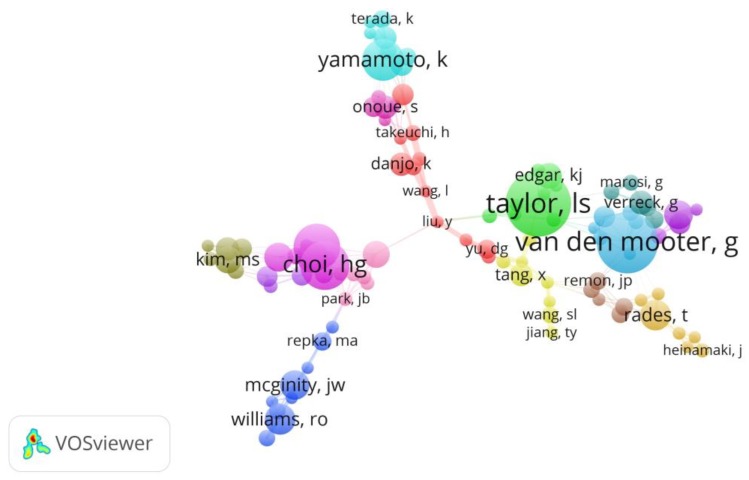
Mapping of authors’ cooperation network. The size of node represents the author’s publication number and the link strength between two nodes means the collaboration intensity between authors.

Figure 7 shows the cooperation among authors in the solid dispersion research area. The size of different color nodes represents the authors’ publication number. Lynne Taylor, a professor at Purdue University, is the leading scientist in solid dispersion field. She has investigated drug-polymer molecular interactions since 1997 [37]. The second scientist in publication number is Guy Van Den Mooter, a professor at University of Leuven. He has focused on research into the physico-chemical characterization of solid dispersions [38]. Other outstanding scientists were Han-Gon Choi, from Hanyang University; Kenji Yamanoto, from International Medical Center of Japan and Tang Xing, from Shenyang Pharmaceutical University, etc. These researchers cooperated closely with each other, especially in the same area. This finding was also agreement with the result of Figure 6 and Table 2. 

### 3.4. The Network of Core Journals

**Figure 8:**
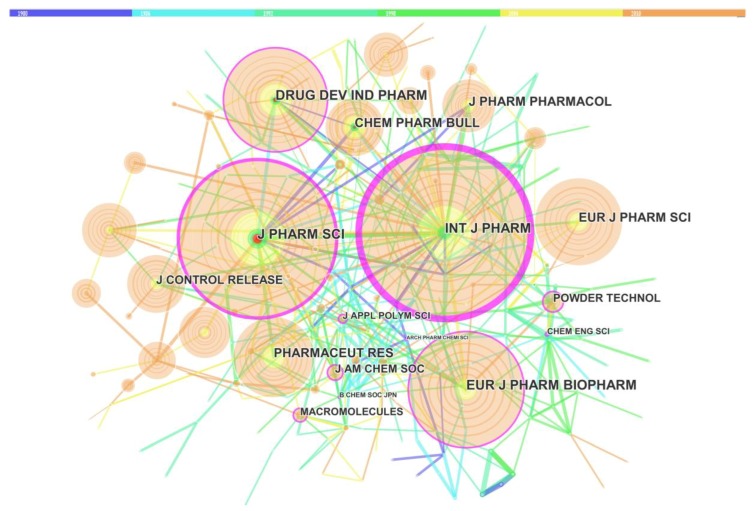
Map of the journal co-citation network. Different colors represent different time slices, deep blue：1980 to1985, sky blue: 1986 to 1991, pink green: 1992 to 1997, green: 1998 to 2003, yellow: 2004 to 2009 and orange: 2010 to 2015. Each circle represents for one journal. The size of circles was proportional to the citation number. The purple ring of the circles indicates the core journals.

Table 4 presents the top 10 journals with the most publications related to solid dispersions. The “core journals” were determined by the journal co-citation analysis technique [39], and the network were described in Figure 8. There were 225 journals and 429 co-citation links among these journals in Figure 8. The top two journals with the most publications were *International Journal of Pharmaceutics* and *Journal of Pharmaceutical Science*, with over 2500 citations. Other highly cited journals, such as *European Journal of Pharmaceutics*, *Biopharmaceutics*, *Drug Development*, and *Industry Pharmacy* also had 2000 citations.

### 3.5. Research Frontiers Trend Shifts 

Research frontier shifts is one of the key factors in a specific research field. The CiteSpace program has two useful functions, keyword burst and co-citation analysis, to analyze the historical directions of research. Keyword burst is an important index for trend analysis in a specific research field within a certain time span. Table 5 indicates 42 burst keywords from 1991 to 2015. Data before 1991 was unavailable because the Web of Science didn’t record keywords in publications before that time. The co-citation analysis is a technique using the cited references as the resource [20,30,40]. Table 6 identifies the cluster terms from co-citation analysis. According to the research topics of solid dispersions, all these keywords or terms could be classified into five categories, including model drugs, carriers, preparation methods, characterization approaches and the mechanism research. The manufacture process and excipients used in the marketed drugs are basically consistent with the results of the literature analysis, as shown in Table 7:

- Model drugs: diazepam, paracetamol, zolpidem, felodipine, sibutramine, kinetisola, itraconazole, ketoprofen, glibenclamide, nifedipine, hydroflumethiazide, griseofulvin, tolbutamide, triamterene, oxazepam, ethenzamide, albendazole, naproxen, temazepam, diflunisal, carbamazepine, rofecoxib, ibuprofen, piroxicam, sulfathiazole, glucosamine, megestrol acetate, tranilast and curcumin;
- Carriers: (1) polymeric carriers: hydroxypropyl methylcellulose, Eudragit, polyethlene glycol, ethyl cellulose, povidone, hydroxypropyl cellulose, poly (ethylene oxide), chitosan, etc.; (2) amphiphilicity carriers: sodium lauryl sulfate, phosphatidylcholine, gelucire 44/14; (3) saccharides carriers: lactose, beta-cyclodextrin.
- Preparation methods: hot melt method, solvent evaporation approach, the spray-drying method and supercritical fluid method.
- Characterization approaches: differential scanning calorimetry, X-ray powder diffraction, atomic force microscopy and NMR.
- Mechanism: controlled release, eutectic mixture, melt agglomeration, amorphous drug stabilization, sustained-release, thermal behavior, dissolution behavior, solid nano-dispersion system, binary dispersion, quantifying drug crystallinity, in vivo drug absorption, drug-release properties, ab initio polymer selection, physical stability studies, amorphous solid dispersion, glassy form, drug-carrier interaction, heterogeneity, excipient distribution, phase diagram, enthalpy relaxation, moisture, miscibility.

## 4. Discussion

According to the characteristics of carriers, solid dispersion could be divided into three generations: the first generation is crystalline carriers, contained urea and sugars [41,42]; The second generation is polymeric carriers, such as polyethylene glycols, polyvinyl pyrrolidone and hydroxyl propyl cellulose, etc. The third generation is surfactants carriers, as Poloxamer, Tween and Gelucire 44/14. The quality of carriers and the solubilization effect of solid dispersion have been improved. For the selection of model drugs, the crystallization tendency and glass-forming ability are two of the important parameters. A solid dispersion may consist of solid solution plus excess solid dispersed within the carrier. Solid dispersion could be achieved by dispersing the “solid solution”, “amorphous form” of “the crystalline drugs” into the carriers or diluents [43]. For example, “glass transition temperature (Tg)”, “thermal behavior”, “phase diagram” and “enthalpy relaxation” were identified as hot terms in Table 5. Moreover, “carrier selection” is of great importance for the formulation development of solid dispersion [44]. Therefore, many related terms were found in Table 5, including “low molecular weight carrier”, “vehicle amphiphilicity”, “ab initio polymer selection”, “drug-carrier interaction”. The selection of a manufacturing process is also very important for amorphous solid dispersion [45,46,47,48]. For example, laboratory-scale preparation preferred melting or solvent-evaporation method due to the efficiency, low cost and fewer types of the materials involved, whereas hot-melting extrusion was popular in the industrial manufacturing process because of the requirement of current Good Manufacturing Practice (cGMP). 

Although the solid dispersion technique had been developed since the 1960s, there are still many key questions that need to be answered. In the recent 10 years, the mechanism of solid dispersions has attracted more and more attention [49,50]. Drug release is an important factor for oral drug bioavailability [51]. The “dissolution behavior” was widely used to predict the “in vivo performance” of a solid dispersion formulation. [52] However, the molecular mechanism of dissolution enhancement of solid dispersion and in vitro-in vivo correlation is still poorly understood. On the other hand, the molecular structure of solid dispersions is still unclear [53,54,55]. Moreover, the molecular structure of solid dispersions strongly relates to their physical stability and formulation development. The “meta-stable state” of drug molecules in the amorphous solid dispersions tends to “recrystallize” to the more stable form during the storage, which is called “physical stability” of solid dispersions [56,57,58,59,60]. The physical aging issue still hinders the development of this formulation technology into commercialization [58,60]. Usually, the test cycle of physical stability needs at least 3-6 months for accelerated stability studies, which is highly time-consuming and costly. Thus, how to shorten the cycle is the key to the formulation development of solid dispersions. 

It is very interesting that computational approaches (e.g., “ab initio polymer selection”) have attracted lots of attention in solid dispersion research. For example, molecular dynamics (MD) simulations not only mimic the structural aspects of systems, but also provide their dynamic and energetic information. Recent studies in our group showed that all-atomic molecular dynamics (MD) simulations with a simulated annealing method were performed to represent molecular models of solid dispersions and mimic the formation process of solid dispersions with hot melt methods [61]. The simulation developed a more reasonable model of amorphous solid dispersions than conventional theory. The study indicates molecular modeling is a powerful technique for the investigation of the molecular mechanism of solid dispersions and the prediction of physical stability of solid dispersions. In addition, integrated cheminformatics and machine learning approaches are also able to fundamentally understand the influencing factors of physical stability and in silico formulation design by the large data sets. The integrated tools may be more predictive and rational to screen the proper carriers and process parameters of solid dispersions. Currently big data prediction of physical stability and dissolution behavior is under way in our laboratory. The modern computational tools are feasible to screen thousands of formulations within a short time that would be impossible to test in the laboratory [62,63,64,65]. 

## 5. Conclusions

This study provided a thoroughly visualized review of the solid dispersion technique and its cooperation network in the recent three decades by science bibliometric mapping approaches. Five topics were analyzed, including model drugs, main excipients, preparation technologies, characterization approaches and the mechanism in solid dispersion researches. Integrated experimental, theoretical and computational tools will benefit in silico formulation design of solid dispersions in the future.

## Figures and Tables

**Table 1:** Global publication shares of the top 10 most productive countries.

Rank	Countries/Territories	TP (%)	TC	ACP
1	USA	633(18.34)	15,096	23.85
2	Japan	383(11.10)	6506	16.99
3	China	370(10.72)	3890	10.51
4	India	349(10.11)	3219	9.22
5	England	289(8.37)	5495	19.01
6	South Korea	212(6.14)	2728	12.87
7	Germany	151(4.38)	3770	24.97
8	Belgium	146(4.23)	3929	26.91
9	Egypt	124(3.59)	808	6.52
10	Italy	118(3.42)	2359	19.99

Note: TP: total number of publications, TC: total citation times, APC: average citation times per paper.

**Table 2:** Productivity and impact of top 20 the most active institutions.

Ranking	Institutes	Country	Records	Average Citation
1	Purdue University	USA	107	26.79
2	Catholic University of Louvain	Belgium	92	34.67
3	Shenyang Pharmaceutical University	China	73	12.96
4	Yeungnam University	South Korea	58	12.98
5	University of Texas Austin	USA	55	32.54
6	University of London	UK	46	17.44
7	Johnson & Johnson	USA	45	37.42
8	Chiba University	Japan	42	12.55
9	University of Copenhagen	Denmark	42	14.83
10	Roche Holding	Swiss	41	18.51
11	Novartis	Swiss	38	23.08
12	Aristotle University of Thessaloniki	Greece	37	30.27
13	Chungnam National University	South Korea	37	23.38
14	Meijo University	Japan	36	18.25
15	University of Greenwich	UK	36	11.06
16	Hanyang University	South Korea	35	8.31
17	AstraZeneca	UK	34	21.12
18	King Saud University	Saudi Arabia	32	5.81
19	Tabriz University MED SCI	Azerbaijan	31	18.94
20	Bristol Myers SQUIBB Co.	USA	30	25.50

**Table 3:** Top 20 global patentees.

Ranking	Assignee Name	Assignee Code	Records
1	Abbott GmbH & Co. KG	ABBO-C	46
2	Reddys Lab Ltd.	REDY-C	20
3	Vertex Pharmaceuticals	VERT-C	17
4	Hetero Research Foundation	HETE-N	16
5	Hanmi Pharmaceutical Co., Ltd.	HANM-C	15
6	Dow Global Technologies LLC	DOWC-C	14
7	Pfizer Inc.	PFIZ-C	13
8	AbbVie Deutschland GmbH & Co. KG	ABBV-N	12
9	AstraZeneca UK	ASTR-C	12
10	Novartis International AG	NOVS-C	12
11	Cadila Healthcare	CDLA-C	11
12	Sandoz AG	SANO-C	11
13	Shenyang Pharmaceutical University	UYSH-N	10
14	Ranbaxy Laboratories Limited	RANB-C	10
15	Sun Yat-Sen University	UYSY-C	10
16	Bend Research	BEND-N	9
17	China Pharmaceutical University	UYCP-C	9
18	Xinjiang Medical University	UYXI-N	8
19	Astellas Pharma US, Inc.	ASTE-C	8
20	Takeda Pharmaceutical Company Limited	TAKE-C	8

**Table 4:** Top 10 active journal and publication number about solid dispersions from 1980 to 2015.

Order	Source Titles	Publication Number
1	*International Journal of Pharmaceutics*	519
2	*Drug Development and Industrial Pharmacy*	303
3	*Journal of Pharmaceutical Sciences*	240
4	*European Journal of Pharmaceutics and Biopharmaceutics*	125
5	*Molecular Pharmaceutics*	121
6	*Chemical Pharmaceutical Bulletin*	119
7	*European Journal of Pharmaceutical Sciences*	107
8	*Pharmaceutical Research*	105
9	*AAPS PharmSciTech*	93
10	*Journal of Pharmacy and Pharmacology*	91

**Table 5:** Keywords burst with time. The time interval was depicted as a blue line, and red line meant the key word burst emergence in this period.

Keywords	Strength	Begin	End	1991–2015
polyethylene glycol	22.4985	1991	2007	▃ ▃ ▃ ▃ ▃ ▃ ▃ ▃ ▃ ▃ ▃ ▃ ▃ ▃ ▃ ▃ ▃ ▃ ▃ ▃ ▃ ▃ ▃ ▃ ▃
nifedipine	17.5424	1991	2006	▃ ▃ ▃ ▃ ▃ ▃ ▃ ▃ ▃ ▃ ▃ ▃ ▃ ▃ ▃ ▃ ▃ ▃ ▃ ▃ ▃ ▃ ▃ ▃ ▃
eudragit	3.6793	1991	2006	▃ ▃ ▃ ▃ ▃ ▃ ▃ ▃ ▃ ▃ ▃ ▃ ▃ ▃ ▃ ▃ ▃ ▃ ▃ ▃ ▃ ▃ ▃ ▃ ▃
hydroflumethiazide	4.7328	1991	2005	▃ ▃ ▃ ▃ ▃ ▃ ▃ ▃ ▃ ▃ ▃ ▃ ▃ ▃ ▃ ▃ ▃ ▃ ▃ ▃ ▃ ▃ ▃ ▃ ▃
coprecipitate	7.7031	1991	2004	▃ ▃ ▃ ▃ ▃ ▃ ▃ ▃ ▃ ▃ ▃ ▃ ▃ ▃ ▃ ▃ ▃ ▃ ▃ ▃ ▃ ▃ ▃ ▃ ▃
differential scanning calorimetry	4.7786	1991	2004	▃ ▃ ▃ ▃ ▃ ▃ ▃ ▃ ▃ ▃ ▃ ▃ ▃ ▃ ▃ ▃ ▃ ▃ ▃ ▃ ▃ ▃ ▃ ▃ ▃
X-ray powder diffraction	4.5573	1991	2004	▃ ▃ ▃ ▃ ▃ ▃ ▃ ▃ ▃ ▃ ▃ ▃ ▃ ▃ ▃ ▃ ▃ ▃ ▃ ▃ ▃ ▃ ▃ ▃ ▃
phosphatidylcholine	8.2027	1991	2001	▃ ▃ ▃ ▃ ▃ ▃ ▃ ▃ ▃ ▃ ▃ ▃ ▃ ▃ ▃ ▃ ▃ ▃ ▃ ▃ ▃ ▃ ▃ ▃ ▃
enteric coating agent	7.4571	1991	2001	▃ ▃ ▃ ▃ ▃ ▃ ▃ ▃ ▃ ▃ ▃ ▃ ▃ ▃ ▃ ▃ ▃ ▃ ▃ ▃ ▃ ▃ ▃ ▃ ▃
griseofulvin	9.8475	1991	1996	▃ ▃ ▃ ▃ ▃ ▃ ▃ ▃ ▃ ▃ ▃ ▃ ▃ ▃ ▃ ▃ ▃ ▃ ▃ ▃ ▃ ▃ ▃ ▃ ▃
sodium dodecyl sulfate	4.486	1991	1996	▃ ▃ ▃ ▃ ▃ ▃ ▃ ▃ ▃ ▃ ▃ ▃ ▃ ▃ ▃ ▃ ▃ ▃ ▃ ▃ ▃ ▃ ▃ ▃ ▃
coevaporate	4.8627	1992	2008	▃ ▃ ▃ ▃ ▃ ▃ ▃ ▃ ▃ ▃ ▃ ▃ ▃ ▃ ▃ ▃ ▃ ▃ ▃ ▃ ▃ ▃ ▃ ▃ ▃
ethyl cellulose	3.7864	1993	2009	▃ ▃ ▃ ▃ ▃ ▃ ▃ ▃ ▃ ▃ ▃ ▃ ▃ ▃ ▃ ▃ ▃ ▃ ▃ ▃ ▃ ▃ ▃ ▃ ▃
tolbutamide	5.6716	1993	2006	▃ ▃ ▃ ▃ ▃ ▃ ▃ ▃ ▃ ▃ ▃ ▃ ▃ ▃ ▃ ▃ ▃ ▃ ▃ ▃ ▃ ▃ ▃ ▃ ▃
triamterene	7.1155	1993	2004	▃ ▃ ▃ ▃ ▃ ▃ ▃ ▃ ▃ ▃ ▃ ▃ ▃ ▃ ▃ ▃ ▃ ▃ ▃ ▃ ▃ ▃ ▃ ▃ ▃
hydroxypropyl cellulose	5.7617	1993	2001	▃ ▃ ▃ ▃ ▃ ▃ ▃ ▃ ▃ ▃ ▃ ▃ ▃ ▃ ▃ ▃ ▃ ▃ ▃ ▃ ▃ ▃ ▃ ▃ ▃
oxazepam	3.6394	1993	1998	▃ ▃ ▃ ▃ ▃ ▃ ▃ ▃ ▃ ▃ ▃ ▃ ▃ ▃ ▃ ▃ ▃ ▃ ▃ ▃ ▃ ▃ ▃ ▃ ▃
ethenzamide	4.8554	1994	2006	▃ ▃ ▃ ▃ ▃ ▃ ▃ ▃ ▃ ▃ ▃ ▃ ▃ ▃ ▃ ▃ ▃ ▃ ▃ ▃ ▃ ▃ ▃ ▃ ▃
poly(ethylene oxide)	3.5901	1995	2007	▃ ▃ ▃ ▃ ▃ ▃ ▃ ▃ ▃ ▃ ▃ ▃ ▃ ▃ ▃ ▃ ▃ ▃ ▃ ▃ ▃ ▃ ▃ ▃ ▃
albendazole	3.5638	1995	2003	▃ ▃ ▃ ▃ ▃ ▃ ▃ ▃ ▃ ▃ ▃ ▃ ▃ ▃ ▃ ▃ ▃ ▃ ▃ ▃ ▃ ▃ ▃ ▃ ▃
Naproxen	4.4423	1996	2006	▃ ▃ ▃ ▃ ▃ ▃ ▃ ▃ ▃ ▃ ▃ ▃ ▃ ▃ ▃ ▃ ▃ ▃ ▃ ▃ ▃ ▃ ▃ ▃ ▃
temazepam	6.899	1998	2008	▃ ▃ ▃ ▃ ▃ ▃ ▃ ▃ ▃ ▃ ▃ ▃ ▃ ▃ ▃ ▃ ▃ ▃ ▃ ▃ ▃ ▃ ▃ ▃ ▃
diflunisal	4.2489	1998	2002	▃ ▃ ▃ ▃ ▃ ▃ ▃ ▃ ▃ ▃ ▃ ▃ ▃ ▃ ▃ ▃ ▃ ▃ ▃ ▃ ▃ ▃ ▃ ▃ ▃
ketoprofen	5.0229	1999	2007	▃ ▃ ▃ ▃ ▃ ▃ ▃ ▃ ▃ ▃ ▃ ▃ ▃ ▃ ▃ ▃ ▃ ▃ ▃ ▃ ▃ ▃ ▃ ▃ ▃
controlled release	4.4497	1999	2002	▃ ▃ ▃ ▃ ▃ ▃ ▃ ▃ ▃ ▃ ▃ ▃ ▃ ▃ ▃ ▃ ▃ ▃ ▃ ▃ ▃ ▃ ▃ ▃ ▃
gelucire 44/14	4.2429	2000	2003	▃ ▃ ▃ ▃ ▃ ▃ ▃ ▃ ▃ ▃ ▃ ▃ ▃ ▃ ▃ ▃ ▃ ▃ ▃ ▃ ▃ ▃ ▃ ▃ ▃
carbamazepine	3.3727	2000	2002	▃ ▃ ▃ ▃ ▃ ▃ ▃ ▃ ▃ ▃ ▃ ▃ ▃ ▃ ▃ ▃ ▃ ▃ ▃ ▃ ▃ ▃ ▃ ▃ ▃
crospovidone	4.1851	2002	2007	▃ ▃ ▃ ▃ ▃ ▃ ▃ ▃ ▃ ▃ ▃ ▃ ▃ ▃ ▃ ▃ ▃ ▃ ▃ ▃ ▃ ▃ ▃ ▃ ▃
supercritical carbon dioxide	6.2183	2003	2009	▃ ▃ ▃ ▃ ▃ ▃ ▃ ▃ ▃ ▃ ▃ ▃ ▃ ▃ ▃ ▃ ▃ ▃ ▃ ▃ ▃ ▃ ▃ ▃ ▃
polyvinylpyrrolidone	7.6429	2004	2009	▃ ▃ ▃ ▃ ▃ ▃ ▃ ▃ ▃ ▃ ▃ ▃ ▃ ▃ ▃ ▃ ▃ ▃ ▃ ▃ ▃ ▃ ▃ ▃ ▃
eutectic mixture	4.0659	2004	2009	▃ ▃ ▃ ▃ ▃ ▃ ▃ ▃ ▃ ▃ ▃ ▃ ▃ ▃ ▃ ▃ ▃ ▃ ▃ ▃ ▃ ▃ ▃ ▃ ▃
rofecoxib	5.144	2004	2007	▃ ▃ ▃ ▃ ▃ ▃ ▃ ▃ ▃ ▃ ▃ ▃ ▃ ▃ ▃ ▃ ▃ ▃ ▃ ▃ ▃ ▃ ▃ ▃ ▃
melt agglomeration	4.9545	2005	2010	▃ ▃ ▃ ▃ ▃ ▃ ▃ ▃ ▃ ▃ ▃ ▃ ▃ ▃ ▃ ▃ ▃ ▃ ▃ ▃ ▃ ▃ ▃ ▃ ▃
microsphere	7.1443	2006	2009	▃ ▃ ▃ ▃ ▃ ▃ ▃ ▃ ▃ ▃ ▃ ▃ ▃ ▃ ▃ ▃ ▃ ▃ ▃ ▃ ▃ ▃ ▃ ▃ ▃
chitosan	4.3876	2006	2009	▃ ▃ ▃ ▃ ▃ ▃ ▃ ▃ ▃ ▃ ▃ ▃ ▃ ▃ ▃ ▃ ▃ ▃ ▃ ▃ ▃ ▃ ▃ ▃ ▃
itraconazole	4.9696	2007	2008	▃ ▃ ▃ ▃ ▃ ▃ ▃ ▃ ▃ ▃ ▃ ▃ ▃ ▃ ▃ ▃ ▃ ▃ ▃ ▃ ▃ ▃ ▃ ▃ ▃
granulation	4.2664	2007	2008	▃ ▃ ▃ ▃ ▃ ▃ ▃ ▃ ▃ ▃ ▃ ▃ ▃ ▃ ▃ ▃ ▃ ▃ ▃ ▃ ▃ ▃ ▃ ▃ ▃
ibuprofen	4.8525	2008	2009	▃ ▃ ▃ ▃ ▃ ▃ ▃ ▃ ▃ ▃ ▃ ▃ ▃ ▃ ▃ ▃ ▃ ▃ ▃ ▃ ▃ ▃ ▃ ▃ ▃
piroxicam	4.126	2009	2010	▃ ▃ ▃ ▃ ▃ ▃ ▃ ▃ ▃ ▃ ▃ ▃ ▃ ▃ ▃ ▃ ▃ ▃ ▃ ▃ ▃ ▃ ▃ ▃ ▃
beta cyclodextrin	4.2647	2010	2011	▃ ▃ ▃ ▃ ▃ ▃ ▃ ▃ ▃ ▃ ▃ ▃ ▃ ▃ ▃ ▃ ▃ ▃ ▃ ▃ ▃ ▃ ▃ ▃ ▃
curcumin	4.7142	2013	2015	▃ ▃ ▃ ▃ ▃ ▃ ▃ ▃ ▃ ▃ ▃ ▃ ▃ ▃ ▃ ▃ ▃ ▃ ▃ ▃ ▃ ▃ ▃ ▃ ▃

**Table 6:** Terms of active research clusters with time from co-citation analysis.

Categories	Year			
	~1980	1980~1996	1997~2003	2004~2010
Model drug	Diflunisal, diazepam	sulfathiazole, paracetamol, zolpidem, glucosamine	sibutramine, tranilast, megestrol acetate	felodipine, kinetisola, itraconazole, ketoprofen, glibenclamide
Carrier	polyethlene glycol 6000, amphiphilicity	sodium lauryl sulfate, hydroxypropyl methylcellulose, eudragit, cellulose derivative matrice, lactose, polyethlene glycol, povidone-sodium, cholate-phospholipid mixed micelle	N/A	Polyvinylpyrrolidone
Preparation method	N/A	spray	supercritical fluid	fusion production, hot-melt extruded, supercritical antisolvent process
Characterizations	N/A	N/A	N/A	thermoanalytical measurement, NMR atomic force microscopy
Mechanism	N/A	amorphous drug stabilization, sustained-release, thermal behavior, dissolution behaviour	crystalline property, solid nano dispersion system	binary dispersion, quantifying drug crystallinity, in vivo drug absorption, drug-release properties, ab initio polymer selection, physical stability studies, acidic decomposition characteristic, free amorphous solid dispersion, glassy form, drug-carrier interaction, heterogeneity, excipient distribution, phase diagram, enthalpy relaxation studies, crystal engineering principle, moisture, miscibility

**Table 7:** Examples of commercially available solid dispersion products.

Order	Products	API	Excipient	Manufacturing Method	Dosage Form	Company
1	Afeditab	Nifedipine	Poloxamer/PVP	Spray drying	Tablet	Elan
2	Afinitor	Everolimus	Hydroxypropyl methylcellulose	Spray dried	Tablet	Novartis
3	Certican	Everolimus	Hydroxypropyl methylcellulose	Spray dried	Tablet	Novartis
4	Cesamet	Nabilone	PVP	-	Tablet	Valeant
5	Crestor	Rosuvastatin	HPMC	Spray drying	Tablet	AstraZeneca
6	Florfenicol	Florfenicol	Enteric cellulose	-	Powder	Hebei Huaqiang
7	Gris-PEG	Griseofulvin	PEG-6000	Melt-extrusion	Tablet	Pedinol
8	Incivek	Teleprevir	HPMCAS-M	Spray drying	Tablet	Vertex
9	Intelence	Etravirin	HPMC	Certican	Tablet	Tibotec
10	Isoptin	Verapamil	HPC/HPMC	Spray drying	Tablet	Abbvie
11	Kaletra	Lopinavir	PVP	Melt extrusion	Capsule	Abbvie
12	Kalydeco	Ivacaftor	HPMCAS	Spray drying	Tablet	Vertex
13	Nivadil	Nivaldipine	HPMC	Spray drying	Tablet	Fujisawa
14	Novir	Ritonavir	PVP	Melt-extrusion	Tablet	Abbott
15	Onmel	Itraconazole	HPMC	Melt-extrusion	Tablet	Sebela
16	Prograf	Tacrolimus	HPMC	Spray drying	Capsule	Fujisawa
17	Rezulin	Troglitazone	HPMC	Spray drying	Tablet	Parke Davis
18	Shuilinjia	Silibinin	Lecithin	-	Capsules	Tianjin Tasly
19	Sporanox	Itraconazole	HPMC	Spray drying on sugar beads	Capsule	Janssen
20	Stivarga	Regorafenib	Povidone K25	-	Tablet	Bayer
21	Votubia	Everolimus	Hydroxypropyl methylcellulose	Spray dried	Tablet	Novartis
22	Zelboraf	Vemurafenib	Hypromellose acetate succinate	Precipitation	Tablet	Roche
23	Zortess	Everolimus	HPMC	Spray drying	Tablet	Novartis
